# Ecological niche comparison and molecular phylogeny segregate the invasive moss species *Campylopus introflexus* (Leucobryaceae, Bryophyta) from its closest relatives

**DOI:** 10.1002/ece3.3301

**Published:** 2017-09-03

**Authors:** Renato Gama, Jesús Aguirre‐Gutiérrez, Michael Stech

**Affiliations:** ^1^ Naturalis Biodiversity Center Leiden The Netherlands; ^2^ Leiden University Leiden The Netherlands; ^3^ Environmental Change Institute School of Geography and the Environment University of Oxford Oxford UK; ^4^ Institute for Biodiversity and Ecosystem Dynamics (IBED) University of Amsterdam Amsterdam The Netherlands

**Keywords:** *Campylopus lamellatus*, *Campylopus pilifer*, incongruence, integrative taxonomy, internal transcribed spacer, morphology

## Abstract

The delimitation of the invasive moss species *Campylopus introflexus* from its closest relative, *Campylopus pilifer*, has been long debated based on morphology. Previous molecular phylogenetic reconstructions based on the nuclear ribosomal internal transcribed spacers (ITS) 1 and 2 showed that *C. pilifer* is split into an Old World and a New World lineage, but remained partly inconclusive concerning the relationships between these two clades and *C. introflexus*. Analyses of an extended ITS dataset displayed statistically supported incongruence between ITS1 and ITS2. ITS1 separates the New World clade of *C. pilifer* from a clade comprising *C. introflexus* and the Old World *C. pilifer*. Ancestral state reconstruction showed that this topology is morphologically supported by differences in the height of the dorsal costal lamellae in leaf cross‐section (despite some overlap). ITS2, in contrast, supports the current morphological species concept, i.e., separating *C. introflexus* from *C. pilifer*, which is morphologically supported by the orientation of the hyaline hair point at leaf apex as well as costal lamellae height. Re‐analysis of published and newly generated plastid *atpB‐rbcL* spacer sequences supported the three ITS lineages. Ecological niche modeling proved a useful approach and showed that all three molecular lineages occupy distinct environmental spaces that are similar, but undoubtedly not equivalent. In line with the ITS1 topology, the *C. pilifer* lineage from the New World occupies the most distinct environmental niche, whereas the niches of Old World *C. pilifer* and *C. introflexus* are very similar. Taking the inferences from ecological niche comparisons, phylogenetics, and morphology together, we conclude that all three molecular lineages represent different taxa that should be recognized as independent species, viz. *C. introflexus*,* C. pilifer* (Old World clade), and the reinstated *C. lamellatus* Mont. (New World clade).

## 1 INTRODUCTION

Accurate species identification is of great importance, for example, in biodiversity assessments, conservation, but also to monitor species with invasive potential. In bryophytes, a morphological species concept is still most commonly employed (Shaw, 2009), but species identification is frequently hampered by relatively few and often (highly) variable morphological characters. This is especially true for species of large and taxonomically complex genera such as *Campylopus* Brid. Although extensive morphological revisions have reduced the number of *Campylopus* species from ca. 1,000 to about 150 (Frahm, 1999; and references therein; Frey & Stech, 2009), the circumscription and identification of many of these species remains difficult. This is especially pressing in delimiting the invasive species *Campylopus introflexus* (Hedw.) Brid. from its closest relatives.

*Campylopus introflexus* is one of the most aggressive invasive moss species (Essl, Steinbauer, Dullinger, Mang, & Moser, 2014) and probably the best known case of moss invasiveness in the world (Carter, 2014; Hassel & Söderström, 2005; Klinck, 2010). Originating from temperate areas of the southern Hemisphere, *C. introflexus* was first recorded outside its native range from Great Britain in 1941 (Richards, 1963) and spread quickly across Europe (Klinck, 2010; Størmer, 1958). Besides Europe, *C. introflexus* was introduced in North America (Carter, 2014). Its negative impact on the biodiversity of natural habitats such as coastal and inland dunes, especially lichen‐rich gray dunes (Essl et al., 2014; Klinck, 2010; Sparrius & Kooijman, 2011), should have brought attention to its troublesome delimitation from closely related species, especially *Campylopus pilifer* Brid. (Frahm, 1974; Frahm & Stech, 2006; Gradstein & Sipman, 1978). Morphologically, *C. introflexus* and *C. pilifer* can be distinguished by the different anatomy of the costa in leaf cross section as well as the orientation of the hyaline hairpoint at leaf apex (Frahm, 1991; Frahm & Stech, 2006; Gama, Faria, Câmara, & Stech, 2016). In *C. pilifer*, the dorsal costal lamellae are two to seven cell rows high and the hairpoint is straight, whereas *C. introflexus* is recognized by lamellae not higher than two cell rows and, when present, reflexed hair points. The effectiveness of using these and other morphological characters to delimit both species have been subject of debate for decades (e.g., Frahm, 1974, 1991; Frahm & Stech, 2006; Gradstein & Sipman, 1978; Stech & Dohrmann, 2004).

Assessing morphological species delimitations in *Campylopus* using molecular data is challenging. Chloroplast markers (*atpB‐rbcL*,* trnT‐F, atpI‐atpH*) provided little phylogenetic signal to delimit species (Stech, 2004), as also reported for other moss genera such as *Dicranum* Hedw. (Lang, Bocksberger, & Stech, 2015), and were partly difficult to sequence, especially *at pB*‐*rbcL*, the slightly more variable of the cpDNA markers. In contrast, the nuclear ribosomal internal transcribed spacer (ITS1‐5.8S‐ITS2) region, the most widely used nuclear marker for plant phylogenetic inferences (Stech & Quandt, 2010), is highly variable in *Campylopus*. Two main types of ITS1 were found, one in *C. introflexus*,* C. pilifer* from the Old World and the sister genus *Pilopogon*, and the other in the New World samples of *C. pilifer* and other *Campylopus* species (discussed in detail by Stech & Dohrmann, 2004). However, the weakly resolved maximum parsimony analyses of ITS1 and ITS2 separately in Stech and Dohrmann (2004) did not allow to fully assess the impact of different phylogenetic signals in both spacers on species relationships in *Campylopus*.

Incongruence between 18S and the internal transcribed spacers was reported by Durand et al. (2002) in the invasive green algal species *Cauler paracemosa* (Forsskål) J. Agardh, and paralogous ITS copies were found in the angiosperm family Calycanthaceae (Li, Ledger, Ward, & del Tredici, 2004); however, incongruence between ITS1 and ITS2, as observed in *Campylopus* has, to the best of our knowledge, not yet been found in any other group of land plants. In contrast to several other widespread *Campylopus* species, *C. introflexus* was shown to be monophyletic and well delimited based on ITS sequences, but its relationships with the two molecular lineages of *C. pilifer* remained ambiguous (Gama et al., 2016; Stech & Dohrmann, 2004; Stech, Sim‐Sim, & Kruijer, 2010; Stech & Wagner, 2005).

As Padial, Miralles, Dela Riva, and Vences (2010) have pointed out, consensus is emerging that species are separately evolving lineages of populations or metapopulations, and the decision that separate lineages should be recognized as distinct species should take into account a combination of different data and analysis methods (integrative taxonomy). Integrative taxonomic approaches combining molecular and morphological evidence have indeed shed new light on species delimitations in bryophytes (e.g., Caparrós, Lara, Draper, Mazimpaka, & Garilleti, 2016; Dirkse, Losada‐Lima, & Stech, 2016; Draper et al., 2015; Medina, Lara, Goffinet, Garilleti, & Mazimpaka, 2012; Sim‐Sim et al., 2017). In *Campylopus,* additional data sources are needed to assess both the molecular phylogenies and the morphological variability. In this study, we use ecological niche comparison techniques (Broennimann et al., 2012) to understand niche differences between the molecular lineages of *C. pilifer* and *C. introflexus*. We consider the niche as describing the set of biotic and abiotic conditions where a species can persist (Grinnell, 1917; Holt, 2009; Hutchinson, 1957), which is the niche concept important for understanding the large‐scale geographic distribution of species (Wiens et al., 2010). According to the competitive exclusion principle, no two species can occupy exactly the same niche space (cannot have equivalent niches) over a long period of time (Gause, 1934). Even in the case of niche conservatism, i.e., when species diversification is not driven by ecological speciation, closely related species tend to be ecologically similar, but not identical (e.g., Kozak & Wiens, 2006; López‐Alvarez et al., 2015). If the ecological niche evolves as part of the speciation process, patterns of ecological differentiation can be potentially useful for species delimitation (Martínez‐Gordillo, Rojas‐Soto, & Espinosa de losMonteros, 2010). In fact, there is growing evidence that ecological niche data can assist species delimitation in different groups of organisms, including vertebrates (e.g., Leaché et al., 2009; Martínez‐Gordillo et al., 2010; Raxworthy, Ingram, Rabibisoa, & Pearson, 2007), invertebrates (e.g., Gurgel‐Gonçalves, Ferreira, Rosa, Bar, & Galvao, 2011; Hawlitschek, Porch, Hendrich, & Balke, 2011), and, most recently, also land plants (e.g., Aguirre‐Gutiérrez, Serna‐Chavez, Villalobos‐Arámbula, Pérez de la Rosa, & Raes, 2015; Shrestha & Zhang, 2015). However, no such study has yet been performed in bryophytes.

Based on the ecological data, molecular analysis of extended datasets (phylogenetic analysis of nuclear ITS and haplotype analysis of plastid *atpB‐rbcL* sequences), and re‐evaluation of diagnostic morphological characters using ancestral state reconstruction, we aim to conclude about species delimitations of *C. introflexus* and *C. pilifer*.

## 2 MATERIALS AND METHODS

### 2.1 Taxon sampling

For molecular phylogenetic analysis, ITS sequences of 89 specimens of *Campylopus* and two of *Pilopogon* Brid. as outgroup representatives, were compiled. Of these, 67 were taken from own previous analyses (Gama et al., 2016; Stech, 2004; Stech & Dohrmann, 2004), three were downloaded from Genbank (one *Campylopus pilifer* and two *C. pilifer* subsp. *vaporarius* (De Not.) Brullo, Privitera & Puglisi, Spagnuolo, Terracciano, Puglisi, & Privitera, 2014), and 21 *C. pilifer* specimens (10 New World clade and 11 Old World clade) were newly sequenced. Fifteen specimens originally identified as other species (*C. arctocarpus* (Hornsch.) Mitt., *C. aureonitens* (Müll. Hal.) A. Jaeger, *C. catarractilis* (Müll. Hal.) Paris, *C. concolor* (Hook.) Brid., *C. incrassatus* Müll. Hal., *C. julaceus* A. Jaeger, *C. pilifer*,* C. richardii* Brid.) were renamed based on the molecular results, except *C. catarractilis* (see Section 4). *AtpB‐rbcL* spacer sequences were compiled for 38 *Campylopus* specimens (20 from Stech, 2004; Stech & Dohrmann, 2004; Frahm & Stech, 2006 and Gama et al., 2016; and 18 newly sequenced). Voucher information and Genbank accession numbers of the newly sequenced specimens are provided in Table 1. For ecological niche comparisons, voucher information from 1,242 additional collections of *C. pilifer* from the New and Old World as well as *C. introflexus* were obtained from different herbaria (L, MO, NY, SP and UB) and from the Global Biodiversity Information Facility (GBIF). Specimens were selected in order to cover the total distribution ranges of the three lineages as far as possible. Data from GBIF collected prior to 1990 were excluded to minimize errors, especially identification problems of older collections, considering that the taxonomic relationships between *C. pilifer* and *C. introflexus* started to be investigated in more detail from that decade onwards (Frahm, 1991, 1999; Frahm & Stech, 2006; Stech & Dohrmann, 2004).

**Table 1:** Voucher information and GenBank accession numbers for the newly generated sequences of *Campylopus* species

Specimen	Original identification	Voucher	Herbarium	Country	Accession number		
					ITS1	ITS2	*atpB‐rbcL*
*C. catarractilis* C089	—	*RSA 106‐09*	L	South Africa	—	—	MF462375
*C. introflexus* C113	—	*Van Zanten 99.06.53*	L	The Netherlands	—	—	MF462377
*C. introflexus* NL257		Gama s.n.	L	The Netherlands	—	—	MF462387
*C. introflexus* RS03	—	*Poloni* et al. *s.n*.	L	Brazil	—	—	KU163137
*C. lamellatus* C225	*C. pilifer*	*Stech* 04‐056	L	Portugal—Madeira	MF416336	MF416357	MF462383
*C. lamellatus* C013	*C. pilifer*	*Greven & Khoeblal 4000/3*	L	Réunion	MF416329	MF416350	MF462372
*C. lamellatus* 1016	*C. pilifer*	*Faria 1016*	UB	Brazil	MF416345	MF416366	—
*C. lamellatus* MG28	*C. pilifer*	*Câmara 2146*	UB	Brazil	MF416346	MF416367	—
*C. lamellatus* SP181	*C. pilifer*	*Soares 1838*	UB	Brazil	MF416349	MF416370	—
*C. lamellatus* MG68	*C. pilifer*	*Gama 176*	UB	Brazil	MF416347	MF416368	—
*C. lamellatus* C156	*C. pilifer*	*Greven & Khoeblal 4000/4*	L	Réunion	MF416332	MF416353	MF462381
*C. lamellatus* Col119	*C. pilifer*	*Linares & Churchill 3608*	MO	Colombia	MF416344	MF416365	—
*C. lamellatus* SP69	*C. pilifer*	*Yano & Kirizawa 32896*	SP	Brazil	MF416348	MF416369	MF462388
*C. lamellatus* C094	*C. pilifer*	*Weigend* et al. *5832*	L	Peru	MF416330	MF416351	MF462376
*C*. *lamellatus* C040	*C. pilifer*	*Frahm s.n*.	L	Brazil	—	—	MF462373
*C. lamellatus* C138	*C. pilifer*	*Allen 6298*	L	USA	—	—	MF462379
*C*. *pilifer* C221	—	*Stech 04‐526a*	L	Portugal—Madeira	MF416334	MF416355	—
*C. pilifer* C127	—	*Müller B816*	L	Equatorial Guinea	MF416331	MF416352	—
*C. pilifer* C231	—	*Frahm M‐9*	L	Portugal—Madeira	MF416337	MF416358	—
*C. pilifer* C236	—	*Luis s.n*.	L	Portugal—Madeira	MF416340	MF416361	—
*C. pilifer* C242	—	*Stech ‐7‐023*	L	Portugal–Madeira	MF416342	MF416363	—
*C. pilifer* C224	—	*Stech 04‐037*	L	Portugal—Madeira	MF416335	MF416356	—
*C. pilifer* C235	—	*Luis s.n*.	L	Portugal—Madeira	MF416339	MF416360	—
*C. pilifer* C241	—	*Stech 07‐008*	L	Portugal—Madeira	MF416341	MF416362	—
*C. pilifer* C211	—	*Frahm s.n*.	L	France	MF416333	MF416354	—
*C. pilifer* C232	—	*Frahm M‐84*	L	Portugal—Madeira	MF416338	MF416359	—
*C. pilifer* C243	—	*Stech 07‐037*	L	Portugal—Madeira	MF416343	MF416364	—
*C. pilifer* C055	—	*Frahm 7611*	L	Rwanda	—	—	MF462374
*C. pilifer* C124	—	*Van Zanten 01.08.01A*	L	The Netherlands	—	—	MF462378
*C. pilifer* C143	—	*Lindlar 458*	L	Cape Verde	—	—	MF462380
*C. pilifer* C248	—	*Stech 07‐73b*	L	Portugal—Madeira	—	—	MF462384
*C. pilifer* C255	—	*Stech 07‐187*	L	Portugal—Madeira	—	—	MF462385
*C. pilifer* C263	—	*Stech 08‐452*	L	Portugal—Madeira	—	—	MF462386

### 2.2 Phylogenetic analysis

Genomic DNA was extracted with the NucleoSpin Plant II Kit (Macherey‐Nagel, Düren, Germany). PCR amplification protocols followed Gama, Stech, Schäfer‐Verwimp, and Peralta (2015). Sequencing was performed by Macrogen, Inc. (www.macrogen.com). Sequences were aligned in Geneious R8 (Kearse et al., 2012). Gaps were treated as informative by simple indel coding (SIC) (Simmons & Ochoterena, 2000) using SeqState (Müller, 2004). No character or position was excluded from the analyses.

Separate phylogenetic analyses of ITS1 and ITS2 were performed under maximum likelihood (ML) and Bayesian inference (BI). The GTR+Γ model, selected under the Akaike information criterion in jModelTest2 (Darriba, Taboada, Doallo, & Posada, 2012; Guindon & Gascuel, 2003), was applied. Maximum likelihood trees were calculated withRaxML version 8.0.26 (Stamatakis, 2014) using raxmlGUI version 1.3.1 (Silvestro & Michalak, 2012). Bootstrap support (BS) values were obtained with a thorough bootstrap algorithm and 10,000 pseudoreplicates. Bayesian analyses were run using MrBayes version 3.2.5 (Ronquist et al., 2012). Bayesian posterior probabilities (PP) were estimated by the Markov Chain Monte Carlo (MCMC) method. Four runs with four chains each (three heated and one cold) were run with 30 × 10^6^ generations, with chains sampled every 1,000th generation and the respective trees written to a tree file. A threshold of <0.01 for the standard deviation of split frequencies was used to assess convergence of runs. Fifty percent majority rule consensus trees and posterior probabilities of clades were calculated combining the four runs using the trees sampled after convergence of the chains and the “burn‐in” (25% of the trees) discarded.

Relationships among *atpB‐rbcL* haplotypes were evaluated based on statistical parsimony, using TCS version 1.21 (Clement, Posada, & Crandall, 2000), with gaps coded as missing data.

### 2.3 Ecological niche comparisons

We selected environmental data regarding eco‐physiological constraints of the target taxa according to recent literature on habitat preferences (Frahm & Stech, 2006; Klinck, 2010; Spagnuolo et al., 2014; Sparrius & Kooijman, 2011; Sparrius, Sevink, & Kooijman, 2012). The selected 10 environmental variables were obtained from WorldClim (www.worldclim.org) and are of high spatial and temporal resolution. The variables are derived from interpolation of weather stations data as described by Hijmans, Cameron, Parra, Jones, and Jarvis (2005) and are listed in Table 2. We included several components of temperature variation and precipitation variables to account for the different biomes within the area of occurrence and its constraints on the survival of the target taxa. All selected variables presented Pearson's correlation ≤0.70 (Dormann et al., 2013) and had a spatial resolution of 10 × 10 km at the equator.

**Table 2:** Environmental variables used for ecological nichemodeling. WorldClim (Hijmans et al., 2005)

Environmental variable
1. Isothermality
2. Temperature Seasonality
3. Maximum temperature of the warmest month
4. Minimum temperature of the coldest month
5. Mean temperature of wettest quarter
6. Mean temperature of driest quarter
7. Precipitation of wettest month
8. Precipitation of driest month
9. Precipitation of driest quarter
10. Precipitation of warmest quarter

We calculated ecological niche characteristics in order to assess how much of the environmental niche space is shared between the molecular lineages of *C. introflexus*,* C. pilifer* from the New World and *C. pilifer* from the Old World. We used an ordination technique with kernel smoothers (Broennimann et al., 2012) to extract the ecological niche space that is occupied by each of the molecular lineages and to quantify niche overlap, equivalence and similarity. The number of occurrences used per molecular lineage may be biased and not representative for the total distribution of the taxa in the environmental space, possibly resulting in an incorrect estimation of their density. Therefore, a kernel density function was applied for smoothing the density of occurrences throughout each cell in the environmental space, leading to a better indication of the suitability of the environmental conditions per lineage. We performed the analyses using a principal component analysis calibrated on the whole environmental space of the study area (PCA‐ent). All analyses were carried out in R (R Development Core Team, 2014).

We obtained the niche breadth of each lineage (amount of ecological niche space available to the different lineages) by using the Levins’ inverse concentration metric (Levins, 1968). To quantify the niches shared by the *Campylopus* lineages, we computed the niche overlap under Schoener's *D* statistic from the ecological niche space (Schoener, 1968; Warren, Glor, & Turelli, 2008), under which the value of *D* ranges from 0 to 1 (0 meaning each two lineages have no overlap in the environmental space and 1 meaning they share the same environmental space).

The niche equivalence test was performed in order to assess whether the ecological niches of each pair of molecular lineages differed significantly from each other or were interchangeable. We compared the niche overlap values (*D*) of the pairs of molecular lineages to a null distribution of 100 overlap values. In case the niche overlap value of the molecular lineages being compared was significantly lower than those acquired by the null distribution (*p *< .05), we assumed the ecological niches not to be equivalent.

Considering that the test for niche equivalency test only assesses whether two species are identical in their niche space by using their exact locations, but disregards the surrounding space, we also performed a niche similarity test. This test assesses whether the ecological niches of any pair of species (in this case, lineages) are more different than would be expected by chance, and considers the differences in the surrounding environmental conditions in the geographic areas where both species are distributed (Warren, Glor, & Turelli, 2010).

We investigated the main environmental variables that constrain the distributions of the lineages based on the loadings of the first two axes of the PCA‐ent.

### 2.4 Ancestral state reconstructions

Two morphological characters that are considered most important to distinguish *C. introflexus* and *C. pilifer*, viz. the hyaline hairpoint at leaf apex and the height of the ventral costal lamellae in leaf cross section, were scored from the molecularly analyzed specimens. Mature leaves were removed from the stems and cross sections made with a razor blade. The highest costal lamella found in cross sections of the upper third of the lamina was scored for each specimen. We distinguished three character states of the hyaline hairpoint (absent, erect, reflexed), whereas for lamella height the measured values, ranging from 1 to 7 cells, were used as character states. Maximum likelihood ancestral state reconstructions of both characters were performed in Mesquite version 3.2 (Maddison & Maddison, 2016) under the one‐parameter Markov k‐state model (Lewis, 2001). Ancestral state reconstructions were based on the topologies of the Bayesian trees and carried out for ITS1 and ITS2 separately. We coded data as missing for the three samples for which ITS sequences were taken from GenBank.

## 3 RESULTS

### 3.1 Phylogenetic analysis

**Figure 1:**
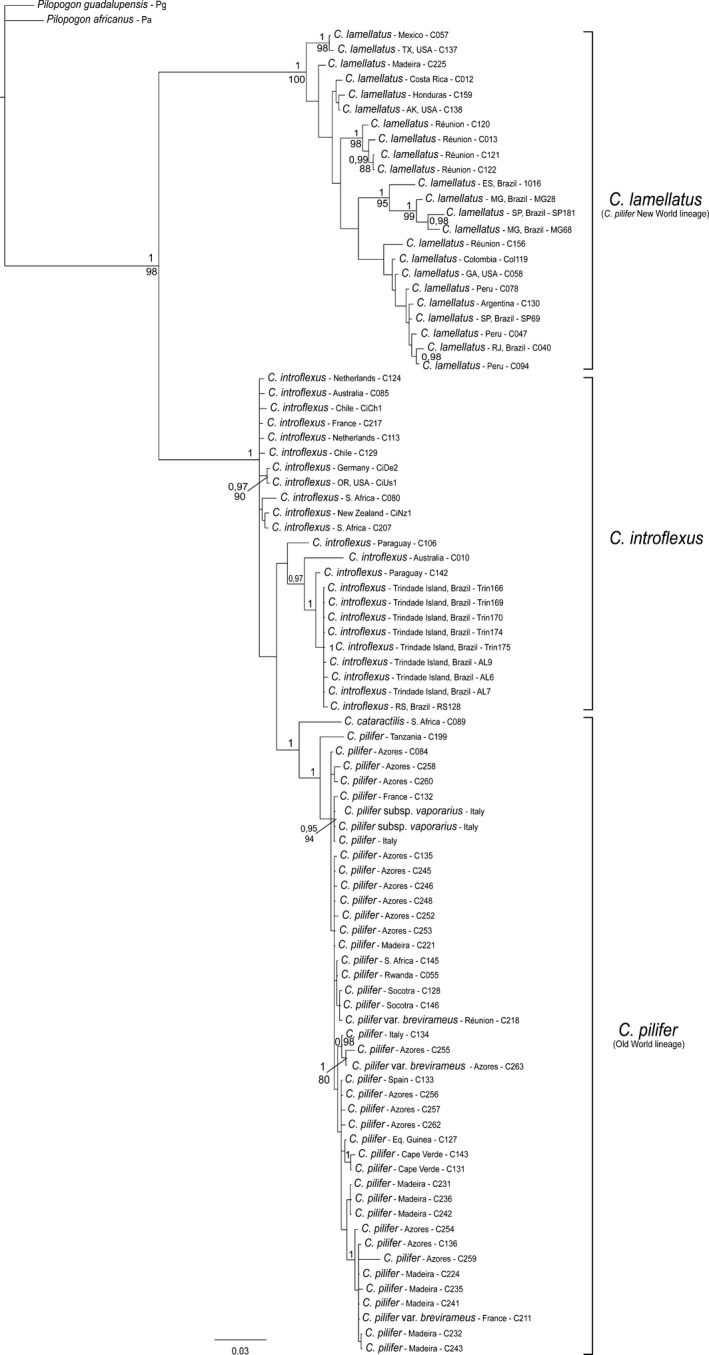
Phylogram obtained from Bayesian analysis of nuclear ribosomal ITS1 sequences, including indels coded by simple indel coding. Values above branches are Bayesian posterior probabilities ≥0.95, values below branches are bootstrap support values ≥75% from maximum likelihood analysis of the same dataset

The ITS1 dataset comprised a total of 1,376 characters (alignment positions 1−1071, indels 1072−1376). The large number of alignment positions was mainly caused by the two main ITS1 types, which for large parts were separated in different blocks in the alignment. The Bayesian inference (BI) consensus tree from ITS1 is shown in Fig. 1, with posterior probabilities (PP) and maximum likelihood bootstrap support values (BS) at the branches. Two major clades were resolved. The first clade had maximum support (PP 1, BS 100%) and comprised the *C. pilifer* samples from the New World (the Americas) plus five samples from Réunion Island and one from Madeira Island. The second clade aggregated *C. pilifer* from the Old World, *C. catarractilis*, and *C. introflexus* with maximum support in the Bayesian analysis (PP 1). *Campylopus introflexus* was resolved as paraphyletic, with a clade of *C. introflexus* samples from mainly South America (Brazil and Paraguay) as sister to the clade of *C. catarractilis*, and the latter sister to *C. pilifer* from the Old World. The Old World *C. pilifer* clade was monophyletic with maximum support.

**Figure 2:**
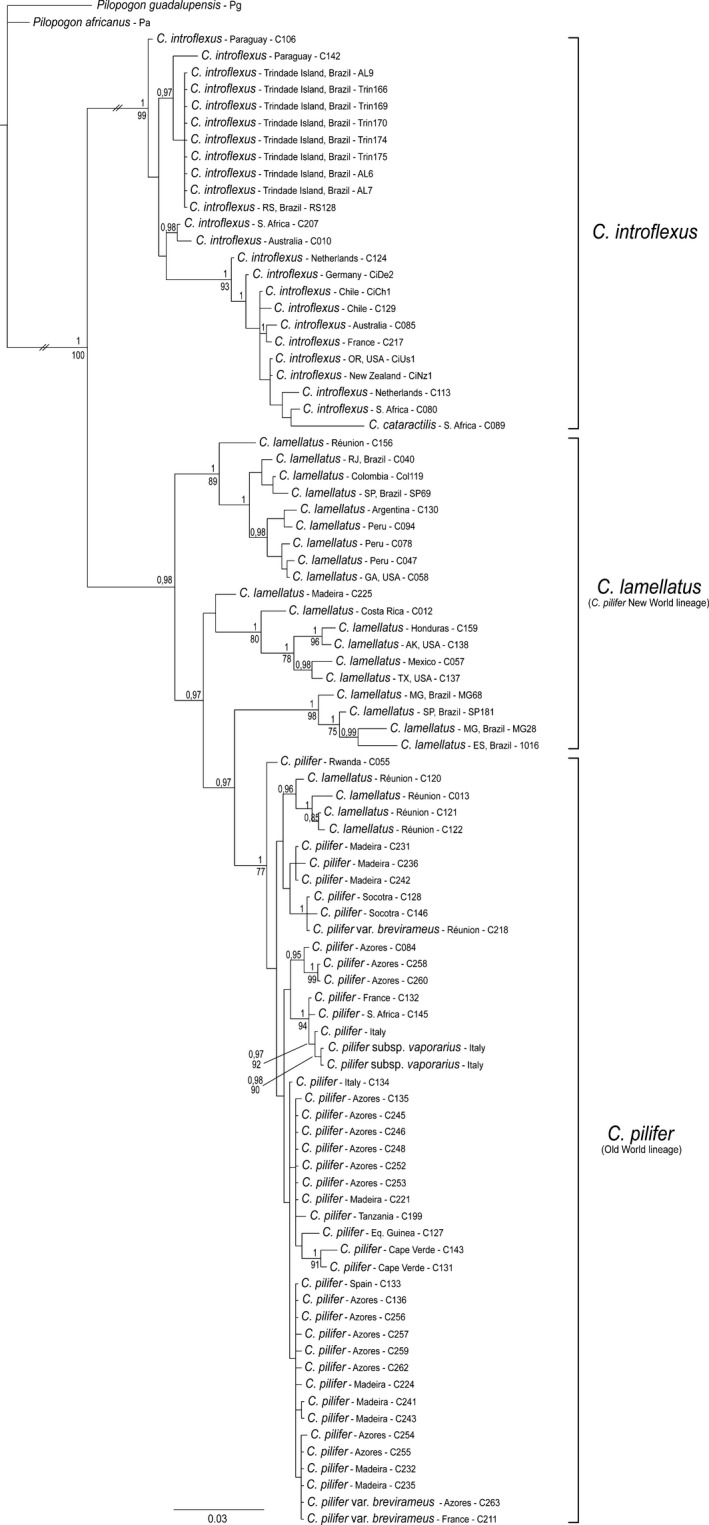
Phylogram obtained from Bayesian analysis of nuclear ribosomal ITS2 sequences, including indels coded by simple indel coding. Values above branches are Bayesian posterior probabilities ≥0.95, values below branches are bootstrap support values ≥75% from maximum likelihood analysis of the same dataset. Branches with the symbol “*//*” were shortened four times

The ITS2 dataset comprised 881 characters (alignment positions 1−655, indels 656−881). The BI consensus tree obtained from ITS2 (Fig. 2) resolved two major clades within *Campylopus*, which corresponded to *C. introflexus* (including *C. catarractilis*, PP 1, BS 99%) and *C. pilifer* s.l. (Old World and New World clades, PP 0.98), respectively. In contrast to ITS1, in the ITS2 analyses, *C. pilifer* from the New World was resolved as paraphyletic to the *C. pilifer* Old World lineage. The latter received a PP of 1 but only moderate BS of 77%, and included four of the five samples from Réunion Island that in ITS1 were part of the New World clade with maximum support.

**Figure 3:**
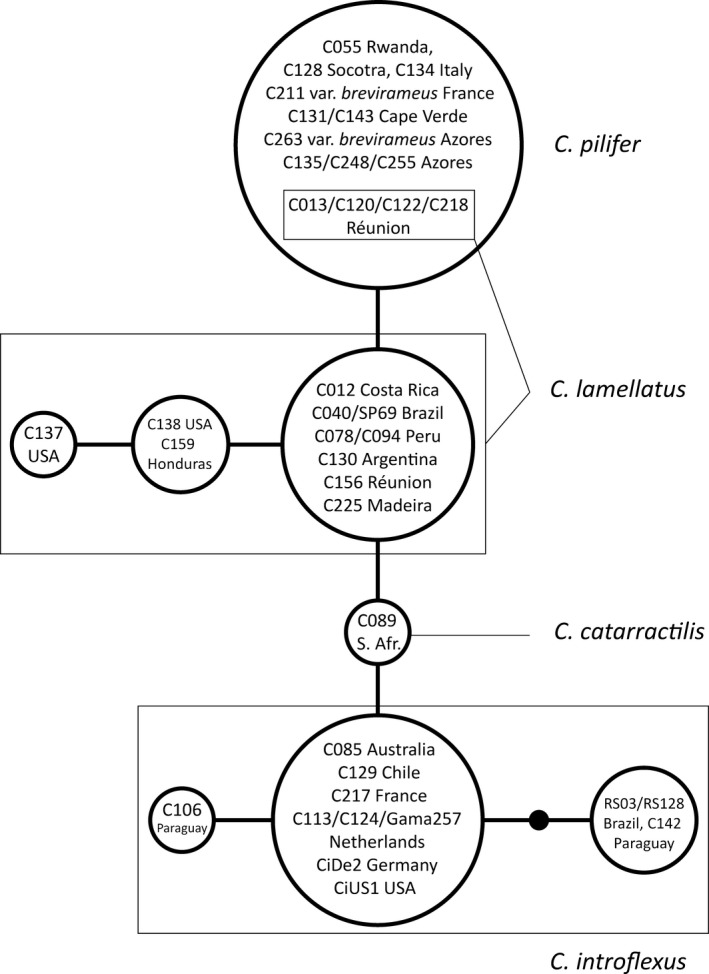
Haplotype network inferred from chloroplast *atpB‐rbcL* spacer sequences using TCS. Circle sizes are an approximate representation of the number of specimens belonging to each haplotype. Squares comprise haplotypes belonging to the same species. The black dot indicates a hypothetical haplotype

All included Old World *C. pilifer* specimens as well as four specimens from Réunion shared the same *atpB‐rbcL* haplotype (Fig. 3). Sequence divergence was higher within *C. introflexus* and the *C. pilifer* from the New World (three haplotypes each). In addition, *C. introflexus* displayed the A‐type loop inversion in the middle part of the spacer, in contrast to the T‐type in *C. pilifer* (cf. details in Stech, 2004), and all three lineages displayed different numbers of AT repeats in a microsatellite region in the spacer.

### 3.2 Ecological niche comparisons

**Figure 4:**
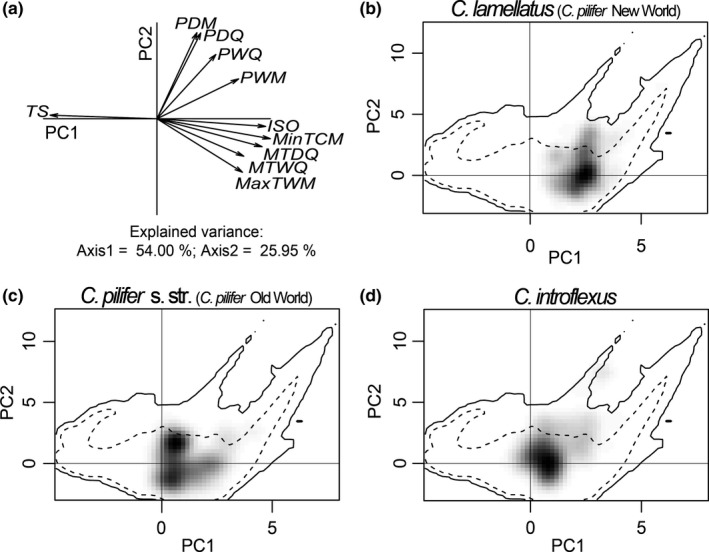
Ecological niches of three distinct molecular lineages of *Campylopus* in environmental space produced by the principal component analysis method (PCA‐ent). The PCA‐ent result represents the climatic niche of the species in the two main axes with the environmental conditions of the complete study area. For each lineage, the gray‐to‐black shading represents the grid cell density of the species’ occurrences (black being the highest density). Dashed and solid lines represent 50% and 100% of the available environment, respectively. (a) PCA with axis 1 explaining 54% of the variance and axis 2 explaining 25.95%. (b) *C. lamellatus*. (c) *C. pilifer*. (d) *C. introflexus*. ISO: Isothermality. MaxTWM, Maximum temperature of warmest month; MinTCM, Minimum temperature of coldest month; MTDQ, mean temperature of driest quarter; MTWQ, mean temperature of wettest quarter; PDM, precipitation of driest month; PDQ, precipitation of driest quarter; PWM, precipitation of wettest month; PWQ, precipitation of warmest quarter; TS, temperature seasonality

The distributions of the three molecular *Campylopus* lineages (*C. introflexus*,* C. pilifer* Old World, *C. pilifer* New World) are related to different responses to the environment and resulted in distinct distributions in niche space (Fig. 4). The analysis of ecological niche properties showed that the two first axes of the PCA‐ent were able to explain 79.95% of the variance of the data. The first axis was determined mostly by temperature‐related bioclimatic factors (namely isothermality, minimum temperature of coldest month, mean temperature of driest quarter, mean temperature of wettest quarter and maximum temperature of warmest month), accounting for 54% of the total variation in environmental conditions for the taxa in the study area (Fig. 4). The highest axis loadings where observed for minimum temperature of coldest month (0.41), isothermality (0.39), and temperature seasonality (0.38). The second axis accounted for 25.95% of the variation and was mainly loaded by precipitation of driest month (0.53), precipitation of driest quarter (0.53) and precipitation of the warmest quarter (0.39). The assessment of niche overlap revealed a small overlap in the environmental space of the three *Campylopus* lineages (Table 3). The *C. pilifer* lineage from the New World occupied the most distinct environmental niche when compared to the other two lineages (Table 3). The pairwise niche similarity comparison between the *C. pilifer* New and Old World lineages indicated that their niche overlap falls within the 95% confidence limits of the null distributions (*p *> .05), leading to non‐rejection of the hypothesis of retained niche similarity (Table 3). The niche similarity between the *C. pilifer* New World clade and *C. introflexus* clade was higher than expected by chance. The niche similarity between the *C. pilifer* Old World lineage and *C. introflexus* was higher than expected by chance in one direction only, indicating that the niche of the Old World clade was more similar than expected by chance to the one of *C. introflexus*, but not vice versa. In contrast, the niche equivalency was rejected for all pairwise comparisons, which indicates that the lineages underwent significant alteration of their distribution in environmental niche space along the process of colonization of the areas within their current distribution.

**Table 3:** Ecological niche comparisons (niche overlap, similarity, and equivalency) for pairwise comparisons of three molecular lineages of *Campylopus* (*C. introflexus*,* C. pilifer* Old World, and *C. pilifer* New World). ‘ns’ not significantly different. The ecological niches can be significantly (*p *< .05) more *similar* or *different* than expected by chance. *p* Values are given between parenthesis

Comparison	Niche overlap (*D*)	Niche similarity a → b	Niche similarity b → a	Niche equivalency
(a) *C. pilifer* New World	0.321	ns	ns	Different
(b) *C. pilifer* Old World		(0.08)	(0.06)	(0.02)
(a) *C. pilifer* New World	0.397	Similar	Similar	Different
(b) *C. introflexus*		(0.02)	(0.04)	(0.02)
(a) *C. pilifer* Old World	0.475	Similar	ns	Different
(b) *C. introflexus*		(0.02)	(0.06)	(0.02)

### 3.3 Ancestral state reconstructions

**Figure 5:**
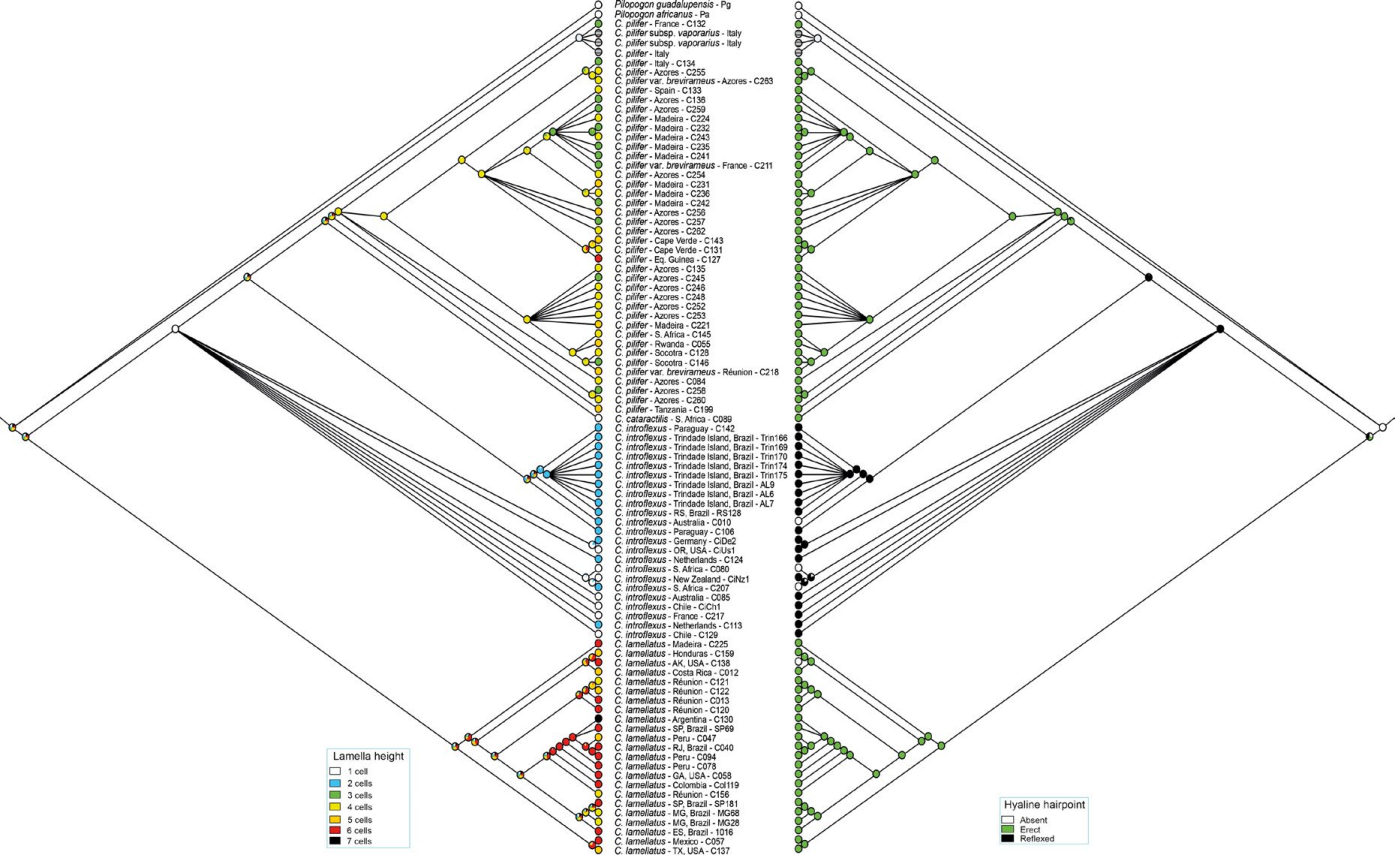
Maximum likelihood ancestral state reconstruction of lamellae height and hairpoint on ITS1

**Figure 6:**
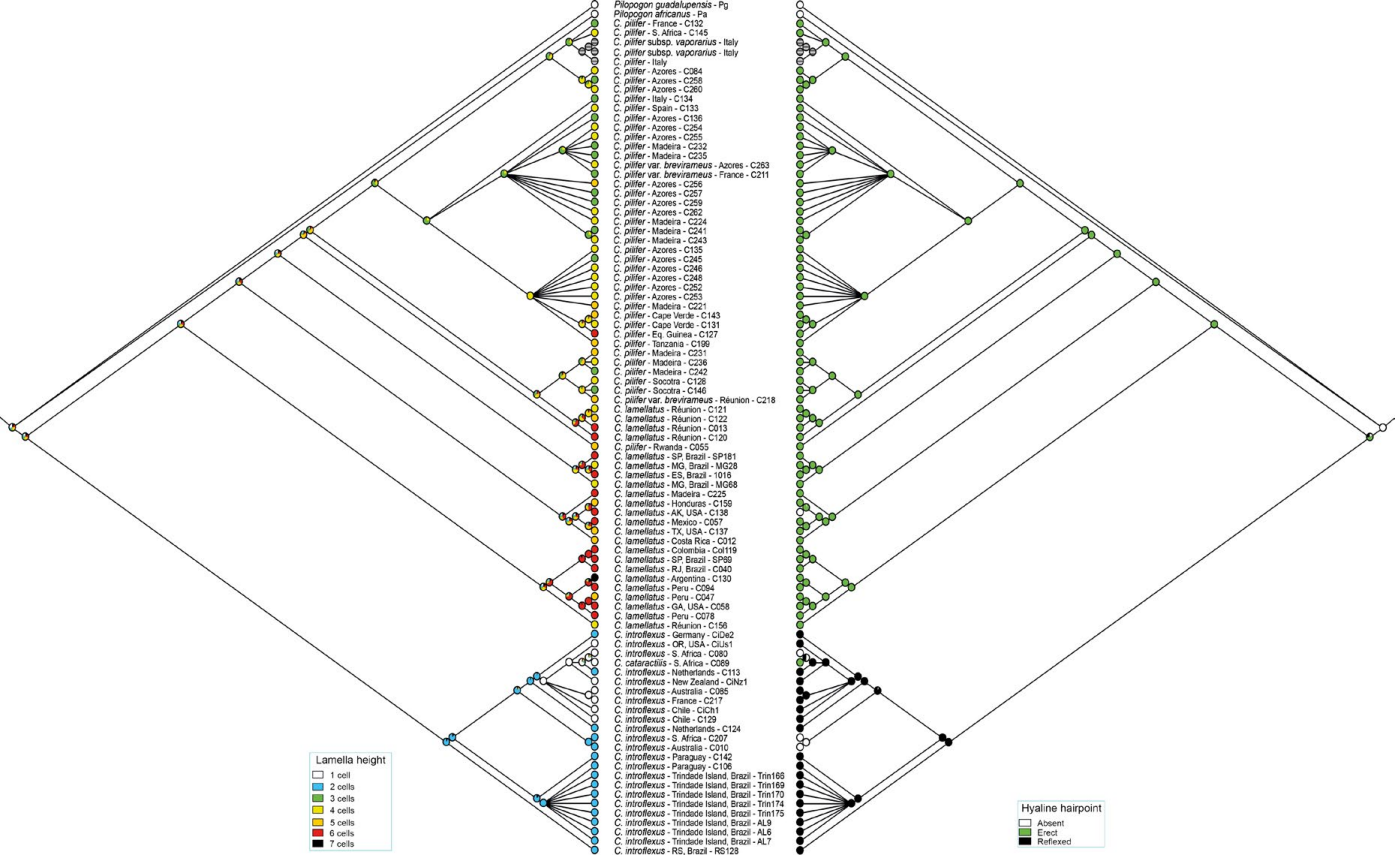
Maximum likelihood ancestral state reconstruction of lamellae height and hairpoint on ITS2

Reflexed hyaline hairpoints were restricted to *C. introflexus*, whereas erect hairpoints occurred in both *C. pilifer* lineages and in *C. catarractilis* (Figs 5 and 6). Apart from the outgroup, the hairpoint was rarely scored as absent, only in three *C. introflexus* specimens as well as a single specimen of *C. pilifer* from the New World. The ITS1 topology suggests that both the reflexed and erect hairpoint states have arisen more than once in the evolutionary history (Fig. 5), whereas in the ITS2 topology the distribution of both character states is in accordance with the current understanding of the species delimitations. Here, with the exception of *C. catarractilis*, the reflexed hyaline hairpoint is resolved as a synapomorphy of *C. introflexus* and the erect hairpoint as a synapomorphy for the two *C. pilifer* lineages. Low costal lamellae (1–2 cell rows high) were restricted to *C. introflexus* and *C. catarractilis* (Figs 5 and 6). In general, the New World clade of *C. pilifer* displayed longer lamellae than the Old World clade. However, homoplasy between the two lineages of *C. pilifer* was observed under the ITS1 topology due to overlapping character states. The ITS2 topology supports a gradual decrease in lamellae height in *C. pilifer*, from long lamellae in the clades branching off first (corresponding to the New World clade) to shorter lamellae in most subclades of the Old World clade.

## 4 DISCUSSION

The model‐based analyses of the present ITS dataset resulted in well‐resolved and supported phylogenetic reconstructions, which allow to assess the different phylogenetic signals of ITS1 and ITS2 in the studied *Campylopus* species more precisely than the maximum parsimony analyses in Stech and Dohrmann (2004). In fact, both internal transcribed spacers display statistically supported incongruence. ITS1 separates the New World clade of *C. pilifer* from a clade comprising *C. introflexus* and the Old World *C. pilifer* (Fig. 1), whereas ITS2 separates *C. introflexus* from *C. pilifer* s.l. (Fig. 2). Further incongruence is observed concerning the positions of *C. catarractilis* as well as four samples from Réunion Island. The ITS2 thus seems to coincide with the current morphological species concept of *C. introflexus* and *C. pilifer* s.l., which is supported by the distribution of reflexed versus straight hairpoints at leaf apex as well as costal lamellae 1−2 versus >2 cell rows high in the ancestral state reconstructions (Fig. 6). The ITS1 topology, however, seems to be supported by morphology as well, separating specimens with costal lamellae (4−)5−6 cell rows high (New World *C. pilifer*) from specimens with mostly lower lamellae, namely 1(−2) cell rows in *C. introflexus* and 3−4(−6) cell rows in Old World *C. pilifer* (Fig. 5).

The presence of conflicting ITS‐based hypotheses and morphological support for each of the ITS lineages, but also the observed overlap in lamellae height at least in some specimens, indicate that it is important to consider other sources of biological information to delimit *Campylopus introflexus* and *C. pilifer*. Despite the smaller number of plastid *atpB‐rbcL* sequences analyzed here and the low resolution of earlier phylogenetic trees based on plastid sequences, the haplotype network approach supports the existence of the three lineages, *C. introflexus*, New World *C. pilifer*, and Old World *C. pilifer* (Fig. 3), without mixing of haplotypes except for the same four samples from Réunion Island that also deviate with ITS. Furthermore, ecological niche comparison proved a useful approach and showed that all three molecular lineages occupy distinct environmental spaces that are similar, but undoubtedly not equivalent (Table 3, Fig. 4). In line with the ITS1 topology, the *C. pilifer* lineage from the New World occupied the most distinct environmental niche, whereas the *C. pilifer* Old World lineage and *C. introflexus* occupied very similar niche spaces.

The inferences from ecological niche comparisons, phylogenetic analyses, and assessment of morphological characters together indicate that all three molecular lineages represent different taxa. We consider the present integrative data sufficient to formally distinguish the three lineages as independent species, viz. *C. introflexus*,* C. pilifer* (Old World clade), and the reinstated species *C. lamellatus* Mont. (New World clade; see Section 5). According to the present data, ITS1 has a discriminatory power to resolve *C. lamellatus*, whereas ITS2 resolves *C. introflexus*, and *C. pilifer* is monophyletic based on both internal transcribed spacers, and the *atpB‐rbcL* spacer may serve as a suitable DNA barcode marker for molecular identification of all three species. However, we acknowledge that the observed patterns and the resulting taxonomic treatment should be tested based on more markers from different genomes in the future. Morphologically, the three species can be distinguished by the combination of hairpoint orientation and height of the dorsal costal lamellae, with *C. introflexus* being most easily recognized, whereas *C. lamellatus* and *C. pilifer* are more overlapping. Ecologically, *C. lamellatus* is most distinct.

As far as the geographical separation of *C. lamellatus* and *C. pilifer* is concerned, already Stech and Dohrmann (2004) and Stech et al. (2010) observed that the former spreads out to oceanic islands of the Old World, namely Madeira Island and Réunion Island. Whereas the specimen from Madeira (C225) and one specimen from Réunion (C156) are part of the *C. lamellatus* clade based on both ITS1 and ITS2 in the present study, the remaining four samples from Réunion Island (C013, C120, C121, C122) share ITS2 sequences with the *C. pilifer* Old World clade. Morphologically, these four samples belong to *C. lamellatus* according to their costal lamellae 5–6 cell rows high. The discrepant phylogenetic results between both internal transcribed spacers suggest that hybridization has possibly occurred between *C. lamellatus* and *C. pilifer*. This is supported by the *atpB‐rbcL* spacer sequences of samples C013, C120, C121, and C122, which all belong to *C. pilifer*. Since the plastid DNA is maternally inherited, *C. pilifer* seems to be the maternal ancestor of the putative hybrid specimen on Réunion. However, further analyses based on a larger taxon and marker sampling is necessary.

Gradstein and Sipman (1978) considered the type specimen of *Campylopus lamellatus* an extreme expression of a general tendency of longer costal lamellae in plants from tropical mountain areas, which had very slender shoots with distinct comal heads, but otherwise fit *C. pilifer*. Consequently, they reduced *C. lamellatus* to a subspecies of *C. pilifer*. Frahm (1985a) assumed a correlation between lamella height and habitat as well, with *C. introflexus* (shorter lamellae) occurring on wetter places than *C. pilifer* (longer lamellae) in regions where both species occur together. Frahm (1985a) furthermore supposed the lamellae in *C. lamellatus* to be longer to enhance gas exchange in rainforests with high temperature and high air humidity. However, the present molecular data indicate that lamellae 5−6 cell rows high have a genetic basis and are not merely modifications due to environmental conditions, at least in *C. lamellatus*. In fact, the specimens of the *C. lamellatus* clade were collected from a broad elevation range (100 to >3,000 m), not only from tropical montane rainforests, but also from very different habitats such as open vegetation with grasses (specimen C120, Réunion), mesquite‐oak savanna (C057, Texas, USA), savanna field among giant *Vellozia* sp. populations (MG68, Minas Gerais, Brazil), *Pinus*‐*Juniperus* forest (C058, Georgia, USA), or the Madeiran laurel forest (C225). In *C. pilifer*, the present data do not indicate a correlation between habitat or geographic area and lamellae height, either. For example, the specimens from the Azores differ considerably in this character (lamellae 3−6 cell rows high). Whether the presence of higher lamellae in both *C. lamellatus* and *C. pilifer* is a result of convergent evolution, possibly as an adaptation to microclimatic conditions, needs further investigation.

Other intraspecific taxa distinguished within the former *C. pilifer* s.l. molecularly clearly belong to the *C. pilifer* Old World clade (Figs 1 and 2), viz. *C. pilifer* var. *brevirameus* (Dix.) J.‐P. Frahm & Stech, which is morphologically closest to *C. introflexus* in its short lamellae, but differs by the erect hair point (Frahm & Stech, 2006), and *C. pilifer* subsp. *vaporarius*, confined to volcanic fumaroles in Italy. The latter has dorsal lamellae 2−3 (rarely 4) cell rows high, and both erect and reflexed hyaline hair points, even on the same stem (Spagnuolo et al., 2014). No molecular data are available yet from *C. pilifer* subsp. *galapagensis* (J.‐P. Frahm & Sipman) J.‐P. Frahm, another narrow endemic described from volcanic rock in Galapagos, which has dorsal lamellae 2−3 cell rows high but differs by the presence of ventral substereids instead of hyalocysts in costa cross section, possible as adaptation to drier habitats (Frahm, 1991).

The present results support recent studies from other groups of organisms indicating that ecological niche comparisons can improve our understanding of the delimitation and relationships of (closely related) species. For example, Aguirre‐Gutiérrez et al. (2015) showed that closely related taxa of *Pinus* subgenus *Strobus* have similar, but not equivalent ecological niches, indicating that they are indeed different species. Shrestha and Zhang (2015) failed to separate taxa of the Huperziaserrata (Thunb.) Trevis. species complex based on morphological data alone. However, using an integrative approach of morphological analysis together with distribution modeling and niche information tests for similarity and equivalency, they were able to circumscribe the different species of the complex. When proposing species circumscriptions of horned lizard (genus *Phrynosoma*) based on molecular phylogenetics, Leaché et al. (2009) retrieved five distinct evolutionary lineages by mtDNA. Nonetheless, when combined with nrDNA, their analyses recovered three lineages, which were further confirmed by morphology and climatic niche models. Similarly, we found that revisiting morphology after the ecological and molecular analyses resulted in a more thorough approach to understand species circumscriptions. In accordance with Raxworthy et al. (2007) and Hawlitschek et al. (2011), we conclude that ecological niche assessments can aid significantly in delimiting species with difficult taxonomic histories, where it can help build a strong case for lumping or splitting species, in combination with other sources of data.

The improved understanding of the delimitations of *C. introflexus*,* C. lamellatus*, and *C. pilifer* is expected to facilitate the identification of collected specimens. This will be particularly helpful to assess the native distribution area of *C. introflexus* and monitor its distribution in areas where it is invasive. With the exception of the *C. catarractilis* specimen, all analyzed specimens originally identified as other *Campylopus* species could be assigned to one of the three species. Similar percentages of misidentified specimens in *Campylopus* (15%, present study) and the *Racomitrium canescens* species complex (20%; Stech et al., 2013) indicate that a percentage of 15%−20% may be expected when analyzing morphologically identified specimens of closely related bryophyte species in an integrative approach.

The African species *Campylopus catarractilis* has not been considered closely related to either *C. introflexus* or *C. pilifer* (cf. Frahm, 1985b). The incongruent position of *C. catarractilis* (sister to *C. pilifer* based on ITS1 and nested in *C. introflexus* based on ITS2) suggested that the sequenced specimen might be misidentified and of hybrid origin similar to the specimens from Réunion discussed above. However, the combination of morphological characters of an erect hairpoint, low lamellae, and the typical serrate leaf apex as diagnostic character for *C. catarractilis* (Frahm, 1985b) in the sequenced specimen, did not allow to unambiguously assign it to either *C. introflexus* or *C. pilifer*. Analysis of further *C. catarractilis* specimens is necessary to infer its taxonomic status.

Despite new insights, further morphological traits should be explored to find additional diagnostic characters that facilitate morphological identification of *C. introflexus*,* C. lamellatus*, and *C. pilifer*. Two observations concerning the costa cross‐section not yet mentioned in the literature were made during this study. Firstly, a group of stereid cells is found above each dorsal lamella intercalated with a larger sub‐stereid cell, except in the center of the leaves where two stereid groups are fused without a sub‐stereid cell between them. This causes the two central lamellae to fuse as well and grow in a V‐shaped orientation. This V‐shaped pattern is more conspicuous when the lamellae are higher and therefore easier to be seen in *C. lamellatus* and *C. pilifer*. Secondly, a marked difference in the delimitation of the costa was observed, which is gradual in *C. pilifer*, most *C. lamellatus*, and *C. introflexus* p.p., but abrupt in a well‐supported clade within *C. introflexus*, a few *C. lamellatus* and in *C. catarractilis*. Despite the fact that these characters do not clearly delimit the molecular lineages, these observations indicate that the morphological and anatomical characters within *Campylopus* are not yet fully employed and that there is potential of novel characters to be found.

Global moss diversity analyses are still hampered by taxonomic and spatial distribution knowledge gaps, particularly in the tropics (Geffert, Frahm, Barthlott, & Mutke, 2013). The impact of misunderstood species delimitations, and misidentifications based on morphology, on species distribution patterns was recently demonstrated for Brazil, where it was understood by the bryological community that no *C. introflexus* occurred in the country, and that all piliferous *Campylopus* specimens belonged to *C. pilifer*. Gama et al. (2016), however, revealed that both species have an overlapping distribution in many places of South America, including Brazil. These findings have immediate impact on the checklist of bryophytes of Brazil, which has not recognized *C. introflexus* yet (Costa et al., 2011). A similar situation may occur in Australia and New Zealand, where currently only *C. introflexus* is reported. Considering the potential distribution of *C. pilifer* and a possible identification bias, it seems likely that *C. pilifer* will be found to occur in Australasia as well, but further investigation is necessary. In accordance with Silva, Vilela, De Marco, and Nemésio (2014), we have shown that ecological niche assessments can aid in the understanding of “data deficient” species.

## 5 TAXONOMIC TREATMENT

*Campylopus lamellatus* Mont. in Ann. Sci. Nat., Bot., sér. 29: 52. 1838.

Type: Bolivia, Chupé, Yungas, d'Orbignys.n. (holotype: PC, isotype: K in BM).

*Campylopus pilifer* var. *lamellatus* (Mont.) Gradst. &Sipman in Bryologist 81: 119. 1978.

*Dicranum lamellatum* (Mont.) Müll. Hal. in Syn. Musc. Frond. 1: 411. 1848.

## ACKNOWLEDGMENTS

We thank CAPES (Brazilian Education agency) for financing the PhD project of the first author as well as the current study. We thank the curators of herbaria MO, NY, SP and UB for loan of specimens.

## CONFLICT OF INTEREST

None declared.
